# Corticosterone levels reflect variation in metabolic rate, independent of ‘stress’

**DOI:** 10.1038/s41598-018-31258-z

**Published:** 2018-08-29

**Authors:** Blanca Jimeno, Michaela Hau, Simon Verhulst

**Affiliations:** 1 Groningen Institute for Evolutionary Life Sciences, University of Groningen, Groningen, The Netherlands; 2 Max Planck Institute for Ornithology, Seewiesen, Germany; 3 University of Konstanz, Konstanz, Germany

## Abstract

Variation in glucocorticoid hormones (GCs) is often interpreted as reflecting ‘stress’, but this interpretation is subject of intense debate. GCs induce gluconeogenesis, and we hypothesized therefore that GC variation can be explained by changes in current and anticipated metabolic rate (MR). Alternatively, GC levels may respond to psychological ‘stress’ over and above its effect on metabolic rate. We tested these hypotheses in captive zebra finches, by inducing an increase in MR using a psychological stressor (noise), and compared its effect on corticosterone (CORT, the primary avian GC) with the effect induced by a decrease in ambient temperature increasing MR to a similar extent. We found the increase in CORT induced by the psychological stressor to be indistinguishable from the level expected based on the noise effect on MR. We further found that a handling and restraint stressor that increased CORT levels also resulted in increased blood glucose levels, corroborating a key assumption underlying our hypothesis. Thus, GC variation primarily reflected variation in energy expenditure, independently of psychological stress. GC levels have many downstream effects besides glucose mobilization, and we propose that these effects can be interpreted as adjustments of physiological functions to the metabolic level at which an organism operates.

## Introduction

Glucocorticoid (GC) hormones (e.g. cortisol, corticosterone) are considered to play a central role in coping strategies and organismal adjustments to environmental variability (reviewed in^1–3^). While variation in GC concentrations and its biomedical and ecological consequences have been the focus of numerous studies (e.g.^4–6^), the causes of variation in GC concentrations are still intensively debated (e.g.^2,3^). Previous work, for example within the field of conservation physiology, often considers elevations in GCs to represent potentially ‘stressful’ conditions^7,8^ (reviewed in^9^; but see^10^). However, ecological and evolutionary physiologists advocated the need to reconsider the causes of GC variation from a wider perspective^2,3,11^. In this context, GCs are also considered as primary mediators of allostasis (i.e. achieving stability through change^3^), integrating physiology and associated behaviours in response or anticipation to changing environments^2,3^. Given this diverse and contentious background, there is a need for a better functional understanding of HPA (hypothalamus-pituitary-adrenal) axis activation.

GCs have many downstream effects on diverse systems, which confound a straightforward interpretation of the HPA axis regulation. A direct effect of GCs is the induction of glucose synthesis in the liver, so that glucose will be released to the blood, making it available for tissues and organs to maintain their metabolism^12,13^. Thus, GCs are expected to fluctuate together with metabolic demands, to provide the resources needed for a perceived (“reactive” response) or anticipated (“anticipatory” response) energy expenditure^11,14^, or to restore glucose levels following an adrenalin supported burst of energy expenditure^11^. Indeed, many studies show that GCs are modulated in parallel with factors that can be assumed to affect energy expenditure. This is true for both predictable (e.g. seasonal or daily temperature variation, breeding, daily activities^2,3,15–20^ and unpredictable and/or uncontrollable (e.g. sudden meteorological events, predator threat, human presence or handling^21–25^); variation in energy demands. However, none of these studies tested whether measurements of manipulated metabolic rate associated with simultaneous measurements of GCs. By using this approach, we recently confirmed a direct relationship between metabolic rate (MR) and GCs in captive zebra finches *Taeniopygia guttata*^26^. The role of GCs in facilitating energy metabolism raises the question whether GC variation can be fully explained as response to current and anticipated variation in MR. Alternatively, “stressors” may increase GCs over and above the response required to meet energetic demands. Because the two alternatives result in very different interpretations of variation in GC levels, this is an important hypothesis to investigate.

We tested whether a psychological stressor induced an increase in corticosterone (CORT, the primary avian GC) levels over and above its effect on MR using noise as psychological stressor in birds housed in metabolic chambers, allowing continuous measurement of MR. This noise effect on CORT was compared with a metabolically induced change in CORT using a decrease in ambient temperature, while in a third (control) treatment category there was no noise or temperature change. The temperature change was tuned to induce on average the same increase in MR as the noise treatment to enable a direct comparison. The temperature change was gradual and within the range routinely experienced by the subjects during that time of year, and induced a gradual increase in MR^26^. For this reason, we consider that only the noise treatment induced a classic stress response in the psychological sense. Both treatments were carried out in the same individuals in a cross-over design, allowing us to compare within-individual CORT responses relative to the control treatment between the two stimuli. Furthermore, as our interpretation of the predicted MR-CORT association is based on the assumption that CORT ensures increasing fuel (i.e. glucose) supply to match higher energetic needs, we also tested whether a response to a standardized stressor^20,27,28^, which induces an increase in plasma CORT, was accompanied by an increase in plasma glucose.

## Materials and Methods

### Subjects

36 birds (18 males and 18 females) were used in this study, all reared at the University of Groningen, the Netherlands, in outdoor aviaries (L × H × W: 310 × 210 × 150 cm) containing 12 pairs each. After reaching independence they were moved to larger single-sex outdoor aviaries (L × H × W: 620 × 420 × 300 cm). One month before the experiment started they were transferred to 4 single-sex outdoor aviaries (L × H × W: 310 × 210 × 150 cm) with 10 birds each (including one extra bird in each aviary as reserve in case of mortality). Food, water and fortified canary food (“egg food”) were always provided *ad libitum*. All birds were born in 2014 and aged 8–13 months when the experiment started.

### Experimental manipulation of metabolic rate and corticosterone sampling

The experiment was carried out in April and May 2015. Each bird went through 4 respirometry sessions of 3 hours each (with a minimum of two weeks between each session), and was subjected to four different treatments in a random order, three of which are included in the present paper: a control treatment under room temperature (22 °C), a ‘cold’ treatment in which ambient temperature was gradually reduced to 12 °C (results were presented in^26^), while the third treatment (subject of the present paper), was an acoustic disturbance (psychological stressor). The fourth treatment, not considered in this paper, consisted of the same noise treatment but in the middle of the three-hour MR measurement period. Average temperature in the outdoor aviaries during sampling hours was 14.38 ± 0.38 °C (mean ± SE) and therefore temperatures used in the experiment fell within the range of temperatures naturally experienced by the subjects during the actual measurement period.

In each respirometry session, two birds (one male and one female) were measured simultaneously, and the sets of two birds remained the same throughout all trials. Birds were captured and transported indoors into the respirometer room in separate cages with access to food, and left undisturbed there for 1 h to acclimate to room temperature (22 °C). After this we took a first blood sample for CORT analysis and birds were weighed to the nearest 0.1 g before being transferred into single metabolic chambers. These first CORT samples are not used in the present paper, but including them in the analyses did not improve the models or change any of the results.

**Figure 1:**
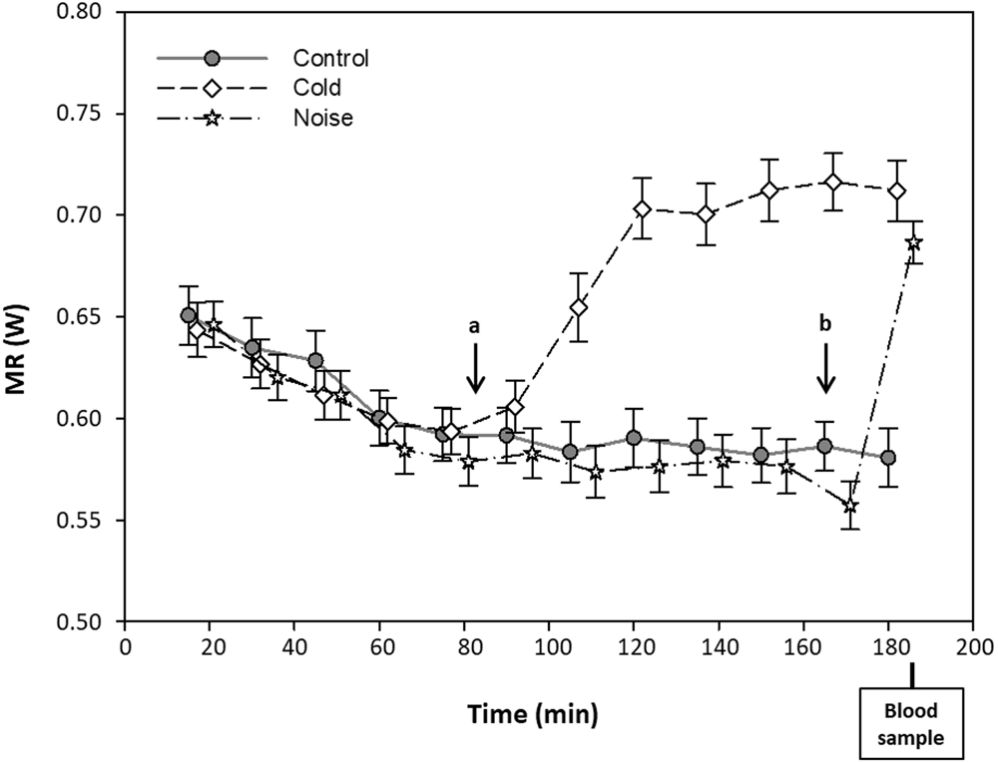
Metabolic rate throughout the trial (3 h) for the control (filled circles, solid line), cold (open diamonds, dashed line) and noise (open stars, dotted line) treatments. Bars represent mean ± SE for each 15 min. interval. The black arrows indicate when the temperature was decreased in the cold treatment (**a**) and when the track started in the noise treatment (**b**).

The respirometer room was then closed and MR measurements started. During the following 1.5 h the birds remained undisturbed, enough time for the MR to reach an approximately stable level (Fig. 1). In the control treatment, the ambient temperature was kept at 22 °C and the birds remained undisturbed for the entire session (3 h). In the cold treatment, after 1.5 hours at 22 °C the ambient temperature was gradually decreased to 12 °C (the target temperature was reached within 15–20 min.; the temperature was changed remotely), and kept at this level for the remaining 1.5 hour. In the noise treatment, temperature was kept at 22 °C and a playlist of sounds/noise was played starting 15 min. before the end of the respirometry session (min. 165–180; Fig. S1). The playlist (first time exposure for all the individuals) consisted of consecutive short tracks (of approximately 30 seconds each) containing sounds of humans (voices, steps, clapping hands) and predators (e.g. sparrow hawk), followed by a 5 min. recording of music (hard-rock). At the end of each measurement session the birds were taken out of the respirometer, a second blood sample was taken and they were weighed again. They were then put into a cage (L × W × H: 40 × 40 × 15 cm) with food and water to recover before being returned to their aviary. All CORT samples were taken within 3 min. after entering the respirometry chamber to minimize disturbance effects on CORT values. We used the average of the two mass measurements as estimate of body mass in the analyses.

Prior to the present study, a pilot study was carried out to adjust both the temperature in the cold treatment and the playlist for the noise treatment to ensure that the cold treatment and the noise exposure induced comparable increases in MR.

MR was measured using an open flow respirometer situated in a climate chamber. Each individual was transferred to a 1.5 L metabolic chamber, without food or water (for technical details see^26,29^). During the measurements, each metabolic chamber or reference outdoor air was sampled every 3 min. for 60 s. In each sampling, we measured O_2_ and CO_2_ concentration and oxygen consumption was calculated using the Eq. of Hill^30^. When analyzing the data we took the average MR during the last 15 min. of the 3 h session as measure of MR for each of the treatments.

### Glucose and corticosterone measurements

A standard restraint protocol^20^ was applied to test whether the CORT increase this induces is accompanied by a proportional increase in blood glucose level. This test was carried out between July 17 and August 5, 2015 using 32 individuals. We sampled two birds per aviary on each day, one in the morning (10:00–11:00) and one in the afternoon (15:00–16:00), to minimize disturbance effects on CORT concentrations. The sequence of aviaries sampled each day was randomized. Immediately after collecting the first sample (baseline CORT) the birds were placed into an opaque cotton bag (restraint stressor), and a second blood sample (stress-induced CORT) was taken after 20 min. In total, no more than 150 μl of blood were taken per individual per day. After blood sampling, birds were put into separate cages with food to recover before being released back into the aviary (usually within 20 min.).

Due to the amount of blood needed for the glucose (90–100 μl) and both CORT traits (40–60 μl), we could measure glucose in only one of the two samples taken per individual on a given day. Hence each individual was subjected to the restraint protocol twice, on different days at least 15 days apart. For each individual, we measured baseline and stress-induced levels CORT on both days, but sampled blood for glucose measurement once on each of the two days, either at baseline or after the restraint treatment. The order in which samples for glucose were taken on the two sampling days (baseline versus stress-induced) was randomized over individuals, balanced by sex and sampling day.

### Blood sampling

Throughout this study, blood samples were taken from the brachial vein, collected in heparinized microcapillary tubes and kept on ice. At the end of each sampling session, plasma for CORT analyses was separated after centrifugation and stored at −20 °C until analyzed. Blood for glucose measurements was immediately taken to the lab for analysis.

### Hormone analyses

Plasma CORT concentrations were measured using an enzyme immunoassay kit (Cat. No. ADI-900-097, ENZO Life Sciences, Lausen, Switzerland), following previously established protocols^20^. Briefly, aliquots of 10 μl plasma along with a buffer blank and two positive controls (at 20 ng/mL) were extracted twice with diethylether. After evaporation, samples were re-dissolved in 280 μl assay buffer. On the next day, two 100 μl duplicates of each sample were added to an assay plate and taken through the assay. Buffer blanks were at or below the assay’s lower detection limit (27 pg/ml). Intra-plate coefficient of variation (CV; mean ± SE) was 10.76 ± 2.77% and inter-plate CV was 8.2% (N = 11 plates; note that plate number was included as a random effect in the statistical analyses). Samples with CV’s > 20% were re-assayed when there was sufficient plasma. Final CORT concentrations were corrected for average loss of sample during extraction, which is 15% in our laboratory^31^.

### Glucose analyses

Plasma glucose concentrations were quantified from whole blood (90–100 µl) with VetScan (Avian/Reptilian Profile Plus reagent rotor; © 2003, Abaxis, Inc., Union City, CA 94587). Glucose detection range for this method is 10–700 mg/dL. Previous tests in our laboratory have shown high repeatabilities between machine trials for the glucose measurements obtained with the VetScan (R = 0.996; N = 12 independent samples; Zuidersma & Verhulst, personal communication).

### Statistical analyses

All statistical analyses were performed using R version 3.3.2 (R Core Team 2016) using either paired t-tests or the function “lmer” of the R package lme4^32^. R^2^ was calculated using the function “r.squaredGLMM” of the R package MuMIn^33^.

#### Metabolic rate and corticosterone

We used paired t-tests within individuals to test for the effect of cold and noise treatments on both MR and CORT. Our subsequent analyses were in two steps in which we compared the MR-CORT association between treatments (temperature decrease vs. noise) using general linear mixed models with individual identity and assay plate (CORT analyses) as random effects. In our first analysis, we focused on absolute CORT levels as the dependent variable. As starting point for the analyses, we used the model we fitted earlier to the temperature treatment data (i.e. the current data set without the noise treatment data^26^), which revealed that there was no discernable temperature effect on CORT when MR and body mass were controlled for. Hence temperature treatment was not included in what we here refer to as the ‘background model’ (including the non-significant temperature treatment as third treatment level did not change the results). MR and CORT showed an accelerating positive relationship which was fitted by including MR squared in the model (as in^26^). The inclusion of sex or its interaction with temperature treatment did not significantly increase the explained variance and were therefore excluded from the background model. The effect of the noise treatment was tested by adding it as a two-level factor (noise vs. no noise) to the background model with or without interaction with MR, and we used Akaike’s Information Criterion^34^ to test whether this improved the model. We initially tested for potential effects of sampling variables on variation in CORT: sampling round (morning/afternoon), sampling order within the pair (1st or 2nd) and whether or not it was the first time the individual was subjected to a respirometry session. However, none of these factors significantly improved the CORT models (Table S1), so we did not include them in further analyses.

In our second analysis we used the within-individual CORT response to the two treatments as dependent variables (i.e., the difference “CORT after cold-CORT after control”, and “CORT after noise - CORT after control”). Treatment and within-individual changes in MR after cold and noise relative to the control condition were used as predictors. Individual identity and assay plate (CORT analyses) were included as random effects.

Although the model fitted for the temperature treatment data only^26^ did not include sex (i.e. there were no sex differences in MR or CORT values, nor in the association between MR and CORT), we tested whether that also held for the noise treatment. Results were consistent with our previous findings, with sex neither explaining variation in the absolute levels (F_1,42.7_ = 1.68; p = 0.20), nor in the response (F_1,31.6_ = 0.93; p = 0.34) models.

CORT levels were logarithmically transformed to normalize the error distribution and CORT change was calculated as ln (CORTcold or noise) – ln (CORTcontrol). Residuals of the final CORT models showed a normal error distribution. One individual male was excluded from the absolute levels analyses because his CORT level was a clear statistical outlier (2.75 times the SD of the model residuals). This individual was however retained in the response analysis, where its residual CORT level was within 1 SD of the model residuals.

#### Glucose and corticosterone

We used paired t-tests using the concentrations of CORT and glucose taken simultaneously (i.e. in the same blood sample) to analyze whether the capture-restraint protocol affected CORT and glucose levels, and subsequently tested for an association between CORT and glucose levels using a mixed linear model with individual identity and assay plate as random factors. The final sample size for the glucose-CORT association was N = 28, because for four individuals it was not possible to obtain one of the two glucose samples. We initially tested for potential effects of sampling variables on variation in glucose: sampling round (morning/afternoon) and glucose sample order (whether the first glucose sample was the baseline or the stress-induced). However, none of them significantly improved the glucose model (Table S2), so we did not include them in further analyses.

### Ethics statement

All experimental procedures were carried out under the approval of the Animal Experimentation Ethical Committee of the University of Groningen, licence 5150 G. All methods were carried out in accordance with these approved guidelines and regulations.

## Results

### Metabolic rate and corticosterone

The noise treatment significantly increased MR compared to the control treatment (t_36_ = −6.00; p < 0.0001; Fig. 1), as did the cold exposure (t_34_ = −5.76; p < 0.0001^26^). As intended, MR levels after noise and cold exposure did not differ significantly, although there was a tendency for MR after cold exposure to be slightly higher (paired t-test; t_35_ = 1.88, p = 0.07), which we attribute to a slight drop in MR just before the noise treatment started (Fig. 1). Likewise, individuals showed higher CORT concentrations after noise (paired t-test: t_36_ = −2.25; p = 0.03) and cold (paired t-test; t_34_ = −2.70; p = 0.011;^26^) exposure compared to the control treatment. CORT levels after cold or noise were indistinguishable (paired t-test: t_35_ = 0.60; p = 0.55).

### Absolute levels analyses

**Figure 2:**
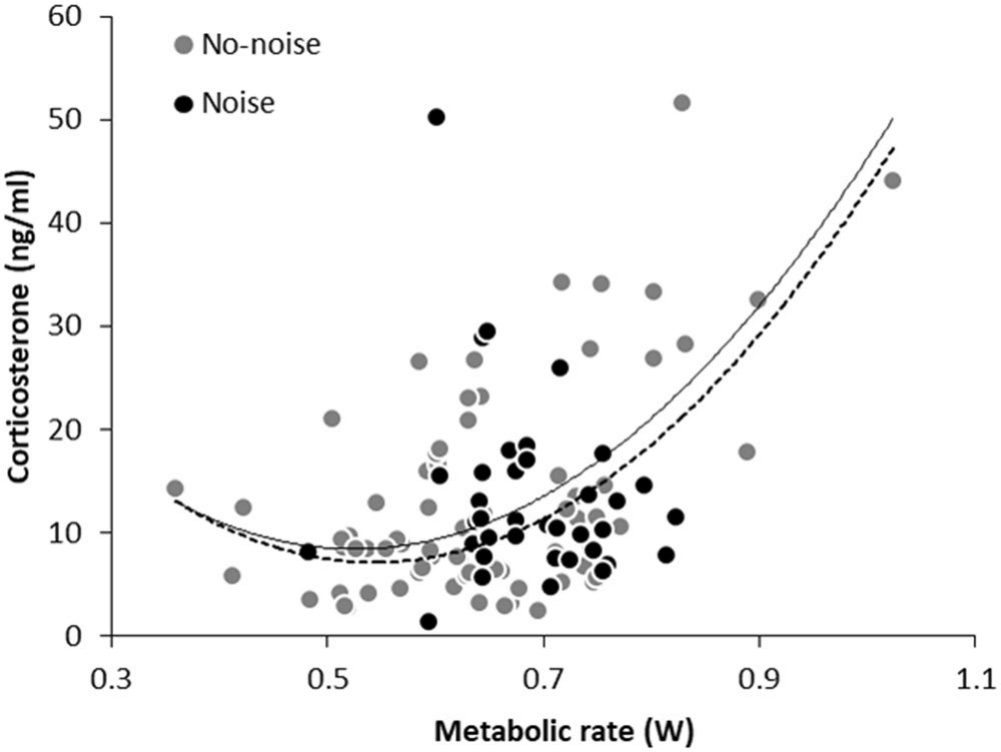
Corticosterone concentration in relation to metabolic rate with (black dots included) and without (grey dots only) noise treatment. The two lines show best fitting regressions calculated with (continuous line) or without (dashed line) the noise treatment as factor in the model. CORT axis is linear, but note that the analyses were carried out using Ln transformed values to normalize the error distribution.

When adding treatment (F_1,71.01_ = 0.40; p = 0.53) to the background model (see methods) this resulted in a model that fitted the data less well (ΔAICc = +1.92), indicating that the relationship between MR and CORT was independent of the noise treatment. Fitting of more complex models, adding e.g. the interaction between MR and treatment, confirmed this finding (Table 1). That the effect of treatment on the association between MR and CORT is negligible is illustrated in Fig. 2, where it can be seen that the fitted lines for models with and without treatment included are almost equal.

**Table 1:** Analyses of absolute CORT concentrations (ng/ml, ln transformed) in relation to body mass and MR (watts).

	**Estimate**	**SE**	**d**.**f**.	**F**	**p**
Intercept	6.058	1.411	103.05		
Body mass	−0.173	0.051	87.53	11.729	**0**.**001**
MR	−6.399	3.703	89.96	2.985	0.087
MR^2^	6.883	2.777	91.46	6.144	**0**.**015**
**Random factors**				**Variance**	
Bird ID				0.131	
Plate				0.000	
Residual				0.232	
**Alternative models**					
			ΔAICc		
Body mass, Treatment, MR, Treatment x MR, MR^2^			1.35		
Body mass, Treatment, MR, MR^2^			1.92		
Body mass, Treatment, MR, Treatment x MR			5.68		
Body mass, Treatment, MR			6.02		
Body mass, Treatment			24.03		
Treatment			38.26		

Final model: marginal R^2^ = 0.288; conditional R^2^ = 0.544. Values in bold indicate *p* < 0.05.

### Response analysis

**Figure 3:**
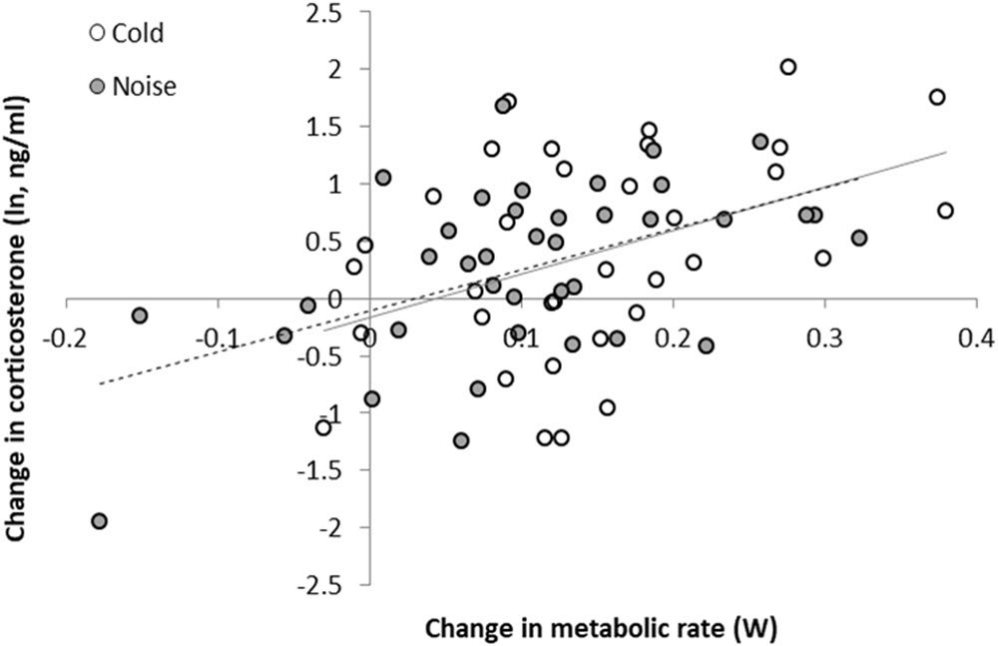
CORT response in relation to MR response under cold (open dots, dashed grey line) and noise (filled dots, continuous grey line). Data shown are changes in MR (x-axis, cold-control/noise-control) and changes in CORT (y-axis, cold-control/noise-control).

Within-individual changes in MR induced by noise were strongly and positively correlated with the changes in CORT (F_1,34_ = 13.12; p < 0.001; Fig. 3), and adding treatment (F_1,34.15_ = 0.091; p = 0.76) or the interaction between treatment and the change in MR to the model did not improve model fits (Table 2). The latter is illustrated in Fig. 3, where it can be seen that the fitted lines for the two treatments are almost equal.

**Table 2:** CORT responses (lnCORT in cold or noise – lnCORT in control) in relation to MR responses to treatments (MR in cold or noise – MR in control).

	Estimate	SE	D.f.	F	p
Intercept	−0.181	0.148	20.63		
MR (change)	4.103	0.792	68.75	26.85	<0.0001
**Random factors**				**Variance**	
Bird ID				0.223	
Plate				0.025	
Residual				0.287	
**Alternative models**					
			ΔAICc		
Treatment, MR (change)			2.29		
Treatment, MR (change), Treatment x MR (change)			4.54		
Treatment			21.13		

Final model: marginal R^2^ = 0.261; conditional R^2^ = 0.602. Values in bold indicate *p* < 0.05.

### Glucose and corticosterone

**Figure 4:**
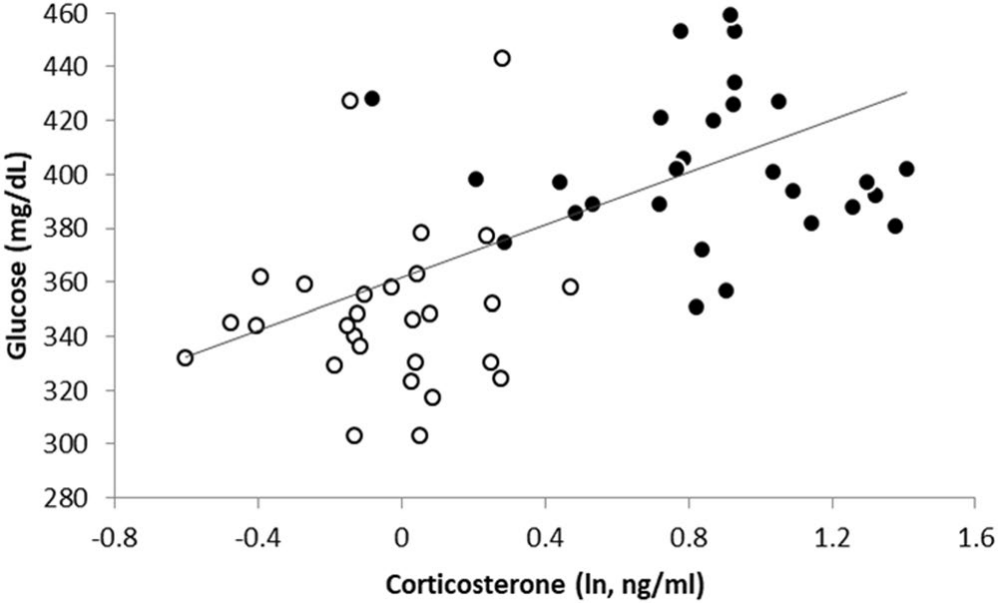
Glucose concentrations in relation to corticosterone concentrations in the same blood sample. Open dots represent baseline levels (within 2 min. after disturbance); black dots represent stress-induced levels (after 20 min. of restraint). Note that baseline and stress-induced samples were taken on different days.

The capture-restraint protocol led to significant increases in CORT (t_28_ = −10.38; p < 0.0001) and glucose (t_28_ = −8.39; p < 0.0001; Fig. S2) concentrations. Likewise, the model testing for the correlation between CORT and glucose concentrations showed a strong association between both traits (F_1,46.8_ = 19.6; p < 0.0001; Fig. 4).

## Discussion

Despite the wealth of information on GCs, the functional interpretation of CORT regulation is still a contentious issue. CORT is often interpreted as indicator of ‘stress’ independent of MR, and we tested this interpretation by comparing CORT between two treatments that both increased MR, with one treatment (noise) inducing a rapid (stress) response, while the other treatment (temperature) induced a gradual homeostatic response. We show that variation in CORT levels was explained by variation in MR, independent of the stimulus that caused the increase in MR. We based our hypothesis on the assumption that CORT induces gluconeogenesis, and this assumption was supported by our finding of a strong association between CORT and blood glucose levels. The latter result is in agreement with previous reports of increases in both plasma GCs (i.e. corticosterone or cortisol) and glucose levels after exposure to stressors in mammals^35^, reptiles^36^ and fish^37,38^. Thus, we propose that the most parsimonious interpretation of our findings is that GC variation is an indicator of variation in MR, independent of the cause of variation in MR. This proposition is supported by the strong association between cortisol (baseline and stress-induced) and MR among mammal species^39^ (but see^40^).

We built our study on the assumption that the noise treatment was perceived as a psychological stressor, while the (gradual) cold treatment was not. Our MR data support this assumption, because the MR increase in response to noise occurred very fast, being already detectable in the first MR measurement after the treatment started. Furthermore, its duration was tightly correlated with the duration of the playlist (our unpublished data), fitting the pattern of an acute response to a stressor^21^. In contrast, the MR response to decreasing temperature was gradual, taking 15–20 min., and occurred in parallel with the decline in ambient temperature (Fig. 1). The proportional response may be due to the fact that the finches were housed in outdoor aviaries between the experiments, and hence the temperature treatment fell within the range of temperature changes that the individuals are exposed to on a daily basis^41^.

Our findings are in agreement with previous comparisons showing similar physiological responses to stimuli of different nature. Harlow and collaborators^23^ found high correlations between simultaneously measured heart rate and plasma cortisol concentrations in sheep exposed to psychological stressors of different intensity. Furthermore, Buwalda and collaborators^42^ showed that an aversive (defeat) vs. rewarding (sex) stimulus both elicited a similar CORT response. These results support the hypothesis that the MR-GC association is independent of the stimuli that trigger MR variation.

Our finding that the MR-CORT association was independent of the cause of MR variation suggests that effects of ‘stressors’ on CORT can be interpreted as indicator of their effect on anticipatory and reactive energetic demands, independent of nature of ‘stressors’. This also implies, conversely, that factors that increase GC levels because they affect MR do not necessarily indicate that they induce ‘stress’ in the psychological sense. From this perspective, we interpret the many downstream effects of GCs^3,13,43^ as allocation adjustments to the metabolic level at which organisms operate, down-regulating functions such as the immune system and reproduction and enhancing learning and memory processes when organism-level metabolic demands are high.

In conclusion, we consider the view that GCs are primarily regulated with respect to their metabolic function the most parsimonious interpretation of our findings, which contrasts with the widespread interpretation of GCs as indicators of ‘stress’. More detailed descriptions of the temporal pattern of MR, GC and glucose levels can provide more general forecasts on the subject. Lastly, we emphasize that through our design we focused our study on within-individual variation, and between-individual variation does not necessarily show the same pattern due to other differences between individuals.

## Electronic supplementary material

Supplementary information

## Data Availability

The data generated during the current study are available from the corresponding author on reasonable request.
