# Supplementary material for: Corticosterone levels reflect variation in metabolic rate, independent of ‘stress’
Source: Sci Rep. 2018 Aug 29;8:13020. doi: 10.1038/s41598-018-31258-z (PMC6115469; doi:10.1038/s41598-018-31258-z)
Supplement: Supplementary file 1 — Supplementary information [file 41598_2018_31258_MOESM1_ESM.pdf]

SUPPLEMENTARY MATERIAL to

"Corticosterone levels reflect variation in metabolic rate, independent of 'stress'"

Blanca Jimeno[1,2], Michaela Hau[2,3] & Simon Verhulst[1]

1. Groningen Institute for Evolutionary Life Sciences, University of Groningen, the Netherlands.
2. Max Planck Institute for Ornithology, Seewiesen, Germany
3. University of Konstanz, Germany.

Table S1: Absolute CORT concentrations (ng/ml, log transformed) in relation to sampling variables. Sampling order (2): individual sampled second; First trial (yes): first respirometer session for that individual. Round (afternoon): individual was sampled in the afternoon.

	Estimate	SE	d.f.	F	p
Intercept	2.263	0.122	44.64		
Sampling order (2)	0.152	0.145	43.19	1.096	0.301
First Trial (yes)	0.105	0.143	85.33	0.534	0.467
Round (afternoon)	0.005	0.149	43.76	0.001	0.972
**Random factors**				Variance	
Bird ID				0.081	
Plate				0.039	
Residual				0.314	

Table S2: Glucose concentrations (mg/dL) in relation to sampling variables. Gluc. order (2): first glucose sample was taken under stress, second in baseline; Round (afternoon): individual was sampled in the afternoon.

	Estimate	SE	d.f.	F	p
Intercept	5.922	0.026	58.00		
Gluc. order (2)	0.055	0.031	58.00	3.173	0.080
Round (afternoon)	-0.034	0.031	58.00	1.174	0.283
**Random factors**				Variance	
Bird ID				0.000	
Plate				0.000	
Residual				0.015	

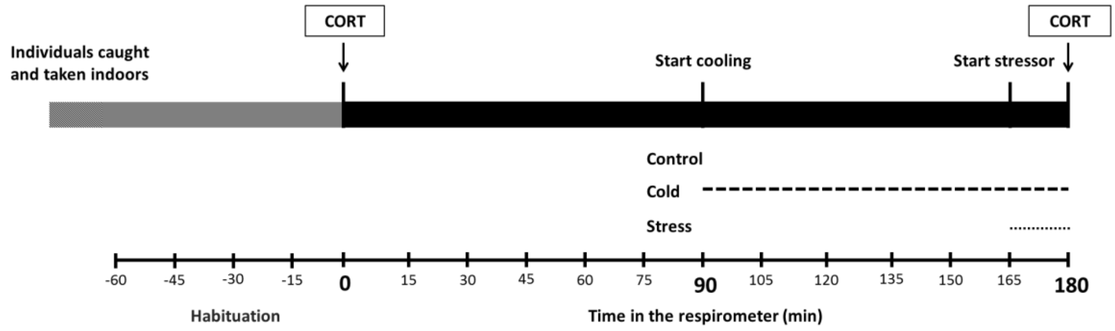

Figure S1: Timeline of the experimental procedure.

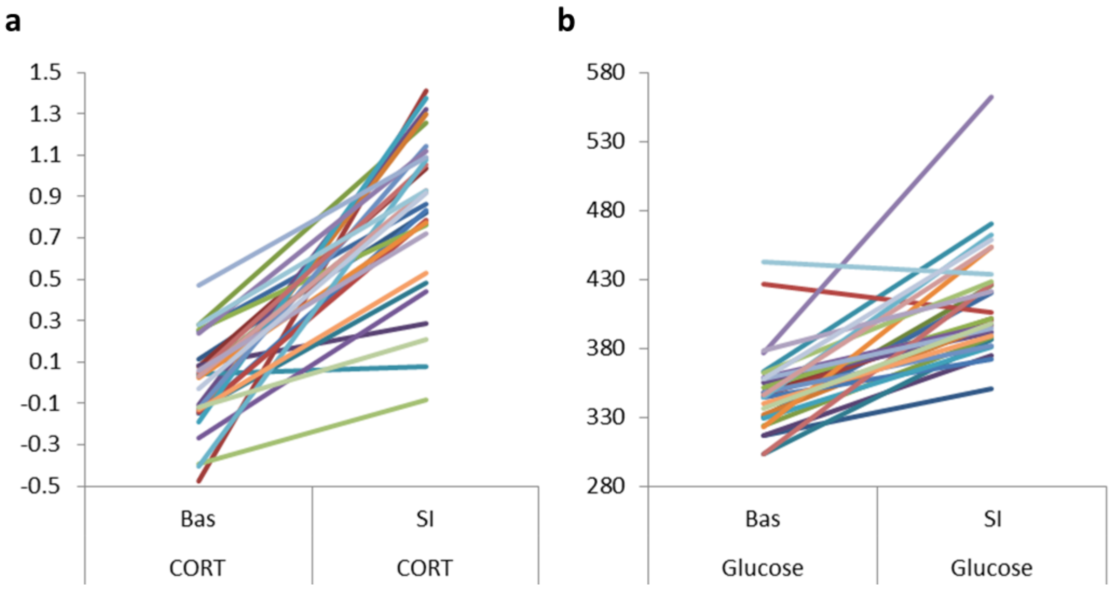

Figure S2: Corticosterone (a, ng/ml) and glucose (b, mg/dl) at baseline (within 2 min. after disturbance) and stress-induced (after 20 min. of restraint). Colors correspond to the same individuals in the two panels.
